# Nipah Virus Detection in *Pteropus hypomelanus* Bats, Central Java, Indonesia

**DOI:** 10.3201/eid3104.241872

**Published:** 2025-04

**Authors:** Dimas Bagus Wicaksono Putro, Arief Mulyono, Esti Rahardianingtyas, Aryo Ardanto, Arum Sih Joharina, Muhammad Choirul Hidajat, Yusnita Mirna Anggraeni, Ristiyanto Ristiyanto, Tika Fiona Sari, N.L.P. Indi Dharmayanti, Triwibowo Ambar Garjito, R. Tedjo Sasmono

**Affiliations:** Environmental Health National Referral Laboratory, Directorate General of Public Health, Ministry of Health of the Republic of Indonesia, Salatiga, Indonesia (D.B.W. Putro, E. Rahardianingtyas, A. Ardanto, A.S. Joharina, T.F. Sari); National Research and Innovation Agency, Bogor, Indonesia (A. Mulyono, M.C. Hidajat, Y.M. Anggraeni, Ristiyanto, N.L.P.I. Dharmayanti, T.A. Garjito, R.T. Sasmono)

**Keywords:** Nipah virus, viruses, zoonoses, vector-borne infections, Pteropus hypomelanus, bats, Central Java, Indonesia

## Abstract

Nipah virus, a zoonotic virus with a high mortality rate, threatens people from Indonesia because of its proximity to affected regions and the presence of bat reservoirs. Molecular screening of 64 *Pteropus hypomelanus* bats in Central Java detected 2 positive bats. Public health authorities should increase surveillance to help prevent human transmission.

Nipah virus, a zoonotic RNA virus member of the genus *Henipavirus*, can cause lethal encephalitis in humans and has an average case-fatality rate of 61.08% (*1*). The 18.2-kb Nipah virus genome encodes 6 structural proteins (N, P, M, F, G, and L) and 3 nonstructural proteins (C, V, and W) (*2*). Nipah virus has 2 genotypes; Malaysia genotype is associated with outbreaks in Southeast Asia, and Bangladesh genotype is prevalent in Bangladesh and India. The 2 genotypes exhibit different transmission dynamics and mortality rates (*3*).

In 1998, a Nipah virus outbreak in Malaysia affected 265 persons, leading to 105 deaths; another outbreak in Singapore in 1999 had 11 cases and 1 death. Pigs (*Sus domesticus*) were the intermediate hosts for both outbreaks (*3*,*4*). In 2014, the Philippines experienced an outbreak involving horses (*Equus caballus*) as intermediate hosts, and human-to-human transmission occurred (*3*,*4*). South Asia has faced recurring outbreaks since 2001, predominantly in Bangladesh and India. In those regions, virus transmission is primarily bat-mediated; *Pteropus* bats serve as natural reservoirs, and human-to-human transmission is also prevalent (*3*,*5*).

*Pteropus* spp. bats are natural hosts of Nipah virus and harbor the virus in saliva, urine, semen, and feces without showing symptoms. The bats are widely distributed in Asia, including Indonesia (*3*). Although no human Nipah virus cases have been reported in Indonesia, its geographic proximity to outbreak regions such as Malaysia and Singapore (*4*) (Appendix Figure) and the widespread presence of *Pteropus* spp. bats (*6*) pose major risks. To assess Nipah virus prevalence, we conducted molecular screening in wild-caught bats in Central Java, Indonesia.

The Health Research Ethics Committee, National Institute of Health Research and Development, Jakarta, Indonesia, approved this study (approval no. LB.02.01/2/KE.691/2021). We collected bats from animal markets in Yogyakarta City and Magelang Regency, Central Java, Indonesia (Appendix Figure), during September 2021 and handled the specimens under strict biosafety protocols in an enhanced Biosafety Level 2+ facility. We collected rectal swab specimens by using the BD universal viral transport system (BD, https://www.bd.com). Morphologic observations and morphometric measurements provided bat identification, as described previously (*7*). For Nipah virus detection, we extracted RNA from rectal swabs by using the RNeasy Mini kit (QIAGEN, https://www.qiagen.com). We conducted reverse transcription PCR (RT-PCR) targeting the nucleocapsid (N) gene, as described previously (*8*). We amplified the gene by heminested PCR using the SuperScript III One-Step RT-PCR kit (Thermo Fisher Scientific, https://www.thermofisher.com). We purified PCR products and sequenced by using the BigDye Terminator v3.1 Kit on a 3500 Genetic Analyzer (Thermo Fisher Scientific). We performed phylogenetic analysis using MEGA 11 software (http://www.megasoftware.net) with a maximum-likelihood algorithm (Appendix).

We obtained 64 fruit bats from traders at animal markets: 37 in Yogyakarta and 27 in Magelang (Figure 1, panels A, B; Appendix). Morphologic identification confirmed all bats as *P. hypomelanus* species (Figure 1, panel B; Appendix Tables 1, 2). RT-PCR of 64 samples detected 2 positive bats from Magelang, Central Java. Sanger sequencing of the N gene confirmed the results and yielded 400-bp and 398-bp sequences. We deposited both sequences in GenBank (accession nos. PQ684035 and PQ684036). 

**Figure 1:**
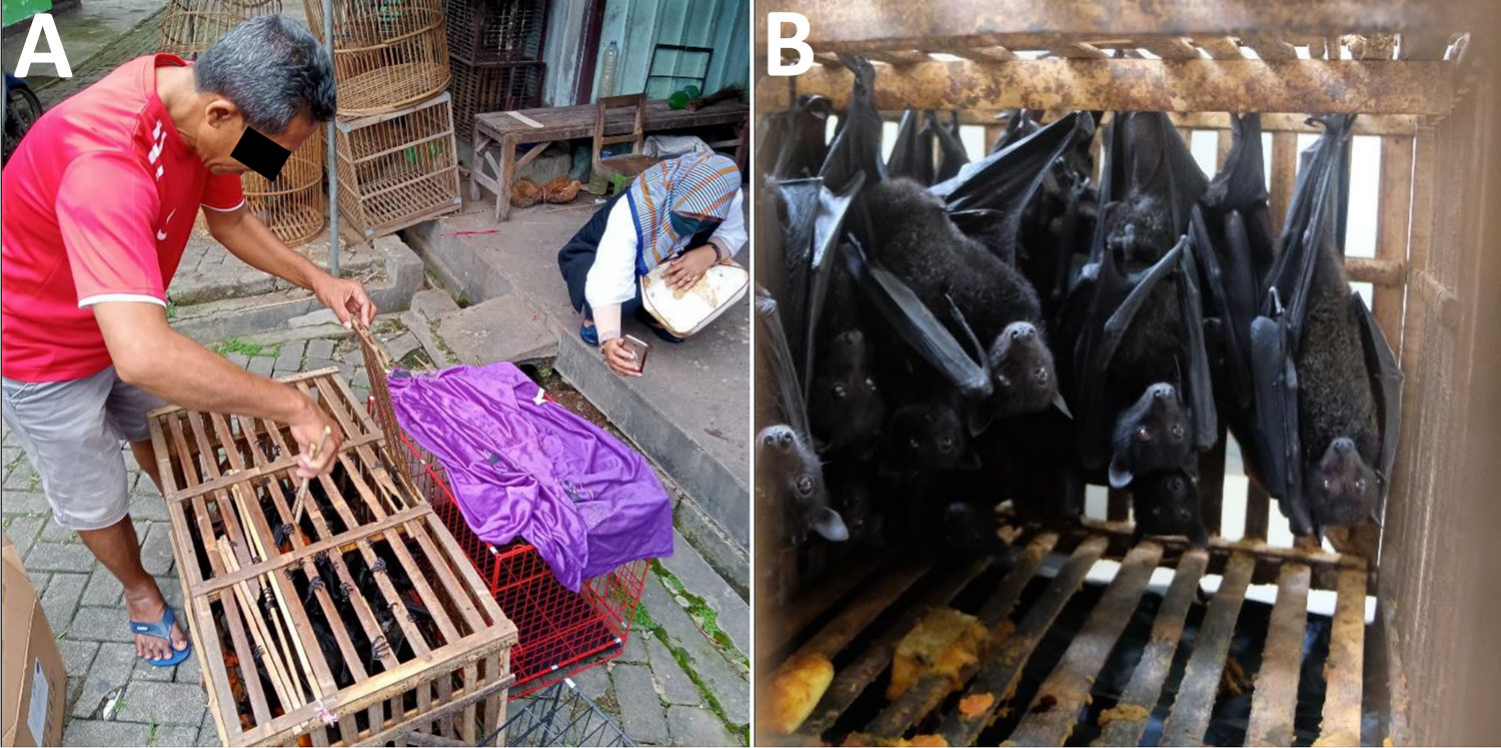
Photographs of bats taken during study of Nipah virus detection in *Pteropus hypomelanus* bats, Central Java, Indonesia. A) Animal trader at local market in Magelang, Central Java, displaying bats for sale. B) Group of *P. hypomelanus* bats hanging inside a wooden cage, sold at an animal market in Magelang, Central Java, Indonesia.

Phylogenetic analysis showed that the isolated Nipah virus in this study belonged to the Malaysia genotype and was closely related to Nipah virus from *P. lylei* bats in Cambodia (99.25% homology with GenBank accession no. KM034755), *P. hypomelanus* bats in Thailand (98.99% homology with GenBank accession no. KT163249), and an isolate from Malaysia (98.74% homology with GenBank accession no. AY029768) (Figure 2). Those findings suggest a strong genetic relationship in Nipah virus in Southeast Asia and indicate regional transmission. The 2 Nipah virus isolates from Indonesia are similar, suggesting local transmission among bats and establishing that viral strain in Indonesia.

**Figure 2:**
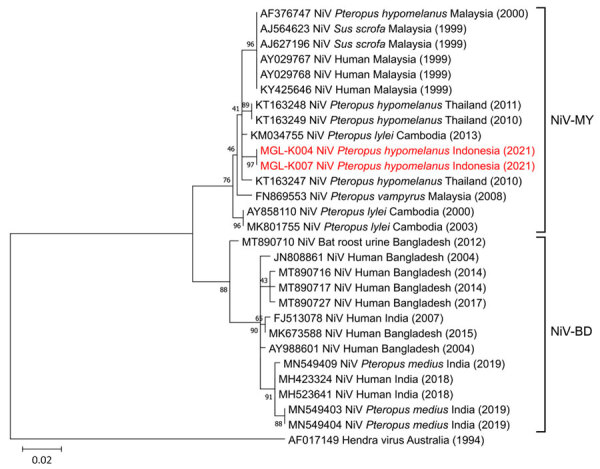
Phylogenetic tree showing NiV detection in *Pteropus hypomelanus* bats in Central Java, Indonesia. Tree illustrates evolutionary relationship of NiV isolates from *Pteropus hypomelanus* bats collected from animal markets in Central Java, Indonesia (specimen codes MGL-K004 and MGL-K007; red font) with strains from Southeast and South Asia (GenBank accession nos., host, and country of isolation indicated). Labels at right illustrates the 2 main genotypes of NiV. Hendra virus was used as an outgroup. The tree was constructed from 400-bp nucleocapsid gene sequences by using the maximum-likelihood algorithm performed in MEGA 11 software (http://www.megasoftware.net). Numbers to the left of the nodes are bootstrap percentages (1,000 replications). Scale bar indicates nucleotide substitutions per site. NiV, Nipah virus; NiV-BD, Bangladesh genotype; NiV-MY, Malaysia genotype.

Nipah virus detection in *P. hypomelanus* bats on the densely populated Java Island represents expansion of the known distribution of that pathogen. Although Indonesia has not experienced Nipah virus outbreaks, Nipah virus has been detected in *P. vampirus* bats in Sumatra Island (*9*). That expanding geographic range confirmed the presence of Nipah virus in various *Pteropus* bats (*3*). Of note, *P. hypomelanus* bats exhibit the broadest distribution in Indonesia (*6*) and are frequently hunted and traded, potentially increasing human–bat interactions and risk for zoonotic transmission. Crowded animal markets with poor sanitation amplify that risk because of virus spillover to humans and domestic animals, as in our study sites.

This study underscores the importance of molecular surveillance of Nipah virus in wildlife reservoirs and humans to identify high-risk areas and transmission pathways. The One Health approach, integrating human, animal, and environmental health, offers a framework for addressing challenges, such as limited surveillance and complex transmission dynamics. By combining data across sectors, stakeholders can enhance their ability to predict and mitigate potential outbreaks (*10*).

In conclusion, our study confirms the detection of Nipah virus in *P. hypomelanus* fruit bats collected from animal markets in Central Java, Indonesia, and provides insight into virus distribution and transmission in the country. Comprehensive surveillance of animal reservoirs is necessary to mitigate human transmission risks and prevent future outbreaks.

Appendix Additional information for Nipah virus detection in *Pteropus hypomelanus* bats, Central Java, Indonesia.
