# Supplementary material for: Nipah Virus Detection in Pteropus hypomelanus Bats, Central Java, Indonesia
Source: Emerg Infect Dis. 2025 Apr;31(4):867–70. doi: 10.3201/eid3104.241872 (PMC11950277; doi:10.3201/eid3104.241872)
Supplement: Appendix — Additional information for Nipah virus detection in Pteropus hypomelanus bats, Central Java, Indonesia. [file 24-1872-Techapp-s1.pdf]

Article DOI: https://doi.org/10.3201/eid3104.241872

EID cannot ensure accessibility for supplementary materials supplied by authors. Readers who have difficulty accessing supplementary content should contact the authors for assistance.

Nipah Virus Detection in *Pteropus hypomelanus* Bats, Central Java, Indonesia

Appendix

Supplemental Methods

Sample Collection

Bats were collected from traders at animal markets in Yogyakarta City, the Special province of Yogyakarta, and an animal market in Magelang Regency, Central Java province, Indonesia (Figure 1), during September 2021. Specimen handling and experimentation were conducted in the Institute for Disease Vector and Reservoir Research and Development (IVRCRD) laboratory at Salatiga, Central Java, Indonesia, following strict biosafety protocols, with all laboratory analyses performed within an enhanced BSL-2+ facility to ensure the safety of personnel and prevent contamination or accidental exposure. Before the rectal swab was taken, the bats were anesthetized by using a combination of ketamine (100 mg/mL) and xylazine (20 mg/mL). Rectal swab collection was performed by using the BD universal viral transport (UVT) system (Becton, Dickinson, and Company). After sample collection, the bats were euthanized following approved animal ethics protocols.

Identification of Bat Species

Bat identification was carried out by morphologic observations (i.e., the presence or absence of claws, hair, and skin membranes between the thighs, tail, and nose/ear shape) and morphometric measurements of the bat's body. Bats were then determined by sex, weight, and measured bat body morphometry (FA = forearm/forearm, Tb = tibia/calf, E = ear/ear, T = tail/tail, HF = hind foot/hind foot) according to methods described previously (*1*,*2*).

Detection Nipah Virus with qRT-PCR

RNA was extracted from rectal swabs from the bats by using the RNeasy Mini kit (QIAGEN, USA), following the manufacturer's instructions. Quantitative reverse transcriptase-

PCR (qRT-PCR) targeting the Nucleocapsid gene was conducted as described by Lo et al. (*3*). The primers and probes used were forward primer (NVBNF2B) 5′-CTGGTCTCTGCAGTTATCACCATCGA-3′; reverse primer (NVBN593R) 5′-ACGTACTTAGCCCATCTTCTAGTTTCA-3′; fluorescent probe (NVBN542P) FAM 5′-CAGCTCCCGACACTGCCGAGGAT-3′ BHQ1. The reagent used in real-time PCR was TaqMan Fast Virus 1-Step Master Mix (Applied Biosystems). The qRT-PCR protocol included a reverse transcription step at 50°C for 5 minutes, followed by predenaturation at 95°C for 20 seconds. The PCR amplification was carried out for 40 cycles with denaturation at 95°C for 30 seconds and annealing at 60°C for 1 minute. Results were monitored in real time, with the threshold cycle (Ct) value assessed. A Ct value <37 indicated a positive result. Positive samples were subsequently amplified by using nested PCR.

Nucleocapsid (N gene) Amplification by Using Hemi-Nested PCR

The initial phase of the hemi-nested PCR involves a 1-step RT-PCR carried out by using the SuperScript III One-Step RT-PCR kit from Invitrogen (Invitrogen, Life Technologies, Carlsbad, USA). The oligonucleotide primers used were as follows: forward primers (NP1F) 5′-CTTGAGCCTATGTATTTCAGAC-3′; reverse primers (NP1R) 5′-GCTTTTGCAGCCAGTCTTG-3′. The thermal cycling conditions were set to begin with a 5-minute denaturation at 94°C, followed by 35 cycles consisting of 30 seconds of denaturation at 94°C, 30 seconds of annealing at 55°C, and 1 minute of elongation at 72°C. The elongation phase was extended by an additional 10 minutes during the final cycle. The subsequent phase was the hemi-nested PCR by using GoTaq Green Master Mix (Promega, Madison, WI, USA). The primers used in that stage were forward primers (NP2F) 5′-CTGCTGCAGTTCAGGAAACATCAG-3′; reverse primers (NP2R) 5′-ACCGGATGTGCTCACAGAACTG-3′. The thermal cycling conditions remain consistent with those used in the first phase. PCR products were then electrophoresed in 2% agarose gel and visualized by SYBR-safe DNA gel staining (Invitrogen, Life Technologies). A 100-bp DNA ladder was used to calculate the PCR product size (*4*).

Nucleocapsid Gene Sequencing and Phylogenetic Analysis

Amplicon PCR products were purified by using Applied Biosystems exoSAP-IT (ThermoFisher Scientific, Vilnius, Lithuania). Cycle sequencing was conducted with the aforementioned primers and an Applied Biosystems BigDye Terminator v.3.1 Cycle Sequencing

Kit (ThermoFisher Scientific, Waltham, MA USA). The resulting cycle sequencing products were purified by using a BigDye Xterminator Purification Kit (ThermoFisher Scientific). Sequence data were obtained through an 3500 Genetic Analyzer and analyzed by using the Sequencing Analysis v5.2 (Applied Biosystems). Sequence alignment and phylogenetic analysis were performed by using MEGA 11 software (*5*) for the maximum-likelihood algorithm with 1,000 bootstrap replicates.

References

1. Jones DP, Kunz TH. *Pteropus hypomelanus*. Mamm Species. 2000;639:1–6. https://doi.org/10.1644/1545-1410(2000)639<0001:PH>2.0.CO;2

2. Suyanto A. Bats in Indonesia [in Indonesian]. Bogor (Indonesia): Puslitbang Biologi-LIPI; 2001.

3. Lo MK, Lowe L, Hummel KB, Sazzad HMS, Gurley ES, Hossain MJ, et al. Characterization of Nipah virus from outbreaks in Bangladesh, 2008-2010. Emerg Infect Dis. 2012;18:248–55. PubMed https://doi.org/10.3201/eid1802.111492

4. Wacharapluesadee S, Hemachudha T. Duplex nested RT-PCR for detection of Nipah virus RNA from urine specimens of bats. J Virol Methods. 2007;141:97–101. PubMed https://doi.org/10.1016/j.jviromet.2006.11.023

5. Tamura K, Dudley J, Nei M, Kumar S. MEGA4: molecular evolutionary genetics analysis (MEGA) software version 4.0. Mol Biol Evol. 2007;24:1596–9. PubMed https://doi.org/10.1093/molbev/msm092

Appendix Table 1. Morphometry of *Pteropus hypomelanus* bats collected from Magelang, Central Java, Indonesia, during Nipah Virus Detection in *Pteropus hypomelanus* Bats in Central Java, Indonesia

				Morphometry, mm					
No.	Specimen code	Age, sex	Weight, g	Head body	Hind foot	Ear	Forearm	Tibia	PCR result
1	MGL-K001	Adult, male	588	260	50	30	168	80	Negative
2	MGL-K002	Adult, male	331	210	45	28	145	70	Negative
3	MGL-K003	Adult, male	429	240	45	29	158	80	Negative
4	MGL-K004	Adult, male	546	255	45	30	170	80	Positive
5	MGL-K005	Adult, male	575	270	45	34	170	85	Negative
6	MGL-K006	Adult, male	522	250	50	30	165	80	Negative
7	MGL-K007	Adult, female	573	260	45	30	180	90	Positive
8	MGL-K008	Adult, male	340	215	45	30	145	65	Negative
9	MGL-K009	Adult, female	404	230	50	31	155	70	Negative
10	MGL-K010	Adult, male	337	220	50	30	140	70	Negative
11	MGL-K011	Adult, male	302	215	45	30	135	70	Negative
12	MGL-K012	Adult, male	619	250	50	30	170	80	Negative
13	MGL-K013	Adult, male	519	250	50	31	170	75	Negative
14	MGL-K014	Adult, male	730	275	45	34	170	80	Negative
15	MGL-K015	Adult, female	424	230	45	35	155	75	Negative
16	MGL-K016	Adult, male	361	220	45	31	150	70	Negative
17	MGL-K017	Adult, female	474	250	50	34	165	75	Negative
18	MGL-K018	Adult, female	436	220	50	30	165	80	Negative
19	MGL-K019	Adult, male	548	260	40	30	165	80	Negative

No.	Specimen code	Age, sex	Weight, g	Morphometry, mm					PCR result
				Head body	Hind foot	Ear	Forearm	Tibia	
20	MGL-K020	Adult, male	454	230	45	32	160	75	Negative
21	MGL-K021	Adult, female	445	260	50	30	160	75	Negative
22	MGL-K022	Adult, female	508	240	45	30	165	75	Negative
23	MGL-K023	Adult, female	372	220	40	30	160	70	Negative
24	MGL-K024	Adult, female	267	200	40	28	130	60	Negative
25	MGL-K025	Adult, male	544	235	50	30	175	85	Negative
26	MGL-K026	Adult, male	329	200	45	30	140	70	Negative
27	MGL-K027	Adult, female	582	280	50	31	175	80	Negative

Appendix Table 2. Morphometry of *Pteropus hypomelanus* bats collected from Yogyakarta, Indonesia, during Nipah Virus Detection in *Pteropus hypomelanus* Bats in Central Java, Indonesia

No.	Specimen code	Age, sex	Weight, g	Morphometry, mm					PCR result
				Head body	Hind foot	Ear	Forearm	Tibia	
1	YK-K001	Adult, female	526	250	32	32	165	89	Negative
2	YK-K002	Adult, male	623	262	45	34	170	89	Negative
3	YK-K003	Adult, female	532	270	40	31	168	83	Negative
4	YK-K004	Adult, female	430	250	40	34	165	86	Negative
5	YK-K005	Adult, female	414	240	45	34	167	89	Negative
6	YK-K006	Adult, male	455	240	50	33	170	80	Negative
7	YK-K007	Adult, male	416	240	45	35	155	80	Negative
8	YK-K008	Adult, female	475	245	50	38	165	85	Negative
9	YK-K009	Adult, female	478	230	45	35	165	85	Negative
10	YK-K010	Adult, female	436	230	45	32	165	85	Negative
11	YK-K011	Adult, female	460	230	45	32	160	80	Negative
12	YK-K012	Adult, male	580	270	55	33	165	80	Negative
13	YK-K013	Adult, female	559	250	50	33	170	80	Negative
14	YK-K014	Adult, female	434	230	40	33	156	78	Negative
15	YK-K015	Adult, female	472	250	40	31	166	81	Negative
16	YK-K016	Adult, male	689	270	45	33	180	85	Negative
17	YK-K017	Adult, female	514	250	45	30	155	80	Negative
18	YK-K018	Adult, female	453	240	40	31	157	80	Negative
19	YK-K019	Adult, male	351	230	40	31	145	75	Negative
20	YK-K020	Adult, female	432	230	45	31	156	85	Negative
21	YK-K021	Adult, female	529	240	50	31	165	80	Negative
22	YK-K022	Adult, male	418	250	50	31	160	80	Negative
23	YK-K023	Adult, male	411	240	50	30	165	85	Negative
24	YK-K024	Adult, female	429	220	45	32	160	79	Negative
25	YK-K025	Adult, female	513	250	45	35	170	80	Negative
26	YK-K026	Adult, female	415	230	50	31	160	75	Negative
27	YK-K027	Adult, female	457	240	50	32	165	90	Negative
28	YK-K028	Adult, female	431	230	40	33	160	75	Negative
29	YK-K029	Adult, female	388	230	45	31	160	75	Negative
30	YK-K030	Adult, female	514	230	50	32	165	80	Negative
31	YK-K031	Adult, female	464	250	50	33	165	80	Negative
32	YK-K032	Adult, female	480	250	50	31	160	80	Negative
33	YK-K033	Adult, female	373	225	45	30	155	80	Negative
34	YK-K034	Adult, female	416	230	50	33	150	75	Negative
35	YK-K035	Adult, male	484	340	50	30	165	85	Negative

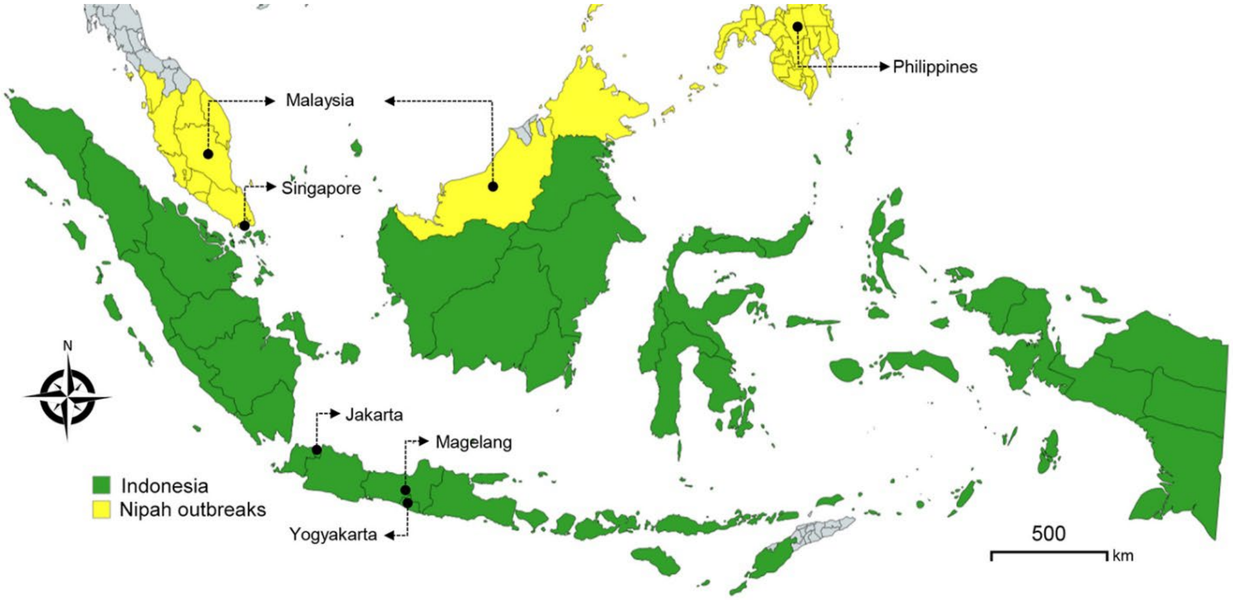

Appendix Figure. Map of Indonesia and the neighboring countries from a study of Nipah virus detection in *Pteropus hypomelanus* bats, Central Java, Indonesia. Yellow indicates locations where Nipah virus has been detected in *P. hypomelanus* bats; green indicates Indonesia. The 2 markets from which Nipah virus–positive bats are located in lower part of map: Magelang and Yogyakarta.
